# Radiological Methods for the Imaging of Congenital Malformations of C6-T1, the First and Second Sternal Ribs and Development of a Classification System, Demonstrated in Warmblood Horses

**DOI:** 10.3390/ani13233732

**Published:** 2023-12-02

**Authors:** Katharina B. Ros, Aldo Doveren, Christie Dreessen, Ralf Pellmann, Francesca Beccati, Elisa Zimmermann, Ottmar Distl

**Affiliations:** 1 Veterinary Clinic PZZ Döhle, 21272 Egestorf, Germany; 2 Veterinary Clinic, 5113 TA Ulicoten, The Netherlands; a.doveren@gmail.com (A.D.); christie@vpgc.nl (C.D.); 3 Veterinary Clinic, 27367 Hellwege, Germany; r.pellmann@rc-pellmann.de; 4 Sports Horse Research Centre, Department of Veterinary Medicine, University of Perugia, 06126 Perugia, Italy; francesca.beccati@unipg.it; 5 Institute for Animal Breeding and Genetics, University of Veterinary Medicine Hannover (Foundation), 30559 Hannover, Germany; elisa.zimmermann@tiho-hannover.de (E.Z.); ottmar.distl@tiho-hannover.de (O.D.)

**Keywords:** horse, cervical radiology, cervical anatomy, cervical spine, cervicothoracic junction, first ribs, ECRM, cervical malformation, rib malformation

## Abstract

**Simple Summary:**

Congenital malformations of the equine cervicothoracic junction have recently been recognized as a finding in performance riding horses. Their clinical relevance is not yet fully understood. To promote better precision in the description of radiological findings among researchers and clinicians, our objective in this study was to identify and grade the equine congenital malformations of C6, C7 and the cranial rib malformations of the first and second sternal ribs using radiographs. Equine cranial rib malformations (ECRMs) refer to cranial rib malformations of the first and second sternal ribs. The radiographic methods described here can help veterinarians to produce radiographs of high diagnostic quality and to assess the severity of malformations according to a uniform system. Future studies will investigate the clinical relevance of different grades of malformations of C6, C7 and the first and second ribs. This knowledge may facilitate improved treatment and management options and more strategic breeding decisions.

**Abstract:**

There are conflicting data in studies on malformations of the cervicothoracic (C-T) junction (C6 to T2, including the first and second ribs), but evidence is mounting that they can be of clinical significance for horses. The objectives of this study were to establish a radiographic protocol for imaging the C-T junction in the field and to classify the radiographic variations found in 39 warmblood horses presented for clinical evaluation due to behavioral or performance issues. Malformations of the ventral lamina of C6 and transposition onto the ventral aspect of C7 were seen in 37/39 (94.9%) horses for both conditions. Rib anomalies were found among the horses with C6 and C7 malformations. A missing first rib, unilateral or bilateral, was found in 3/35 (11.4%) horses, a unilateral shortening of the rib in 17/35 (48.6%) horses, a bilateral shortening of the ribs in 12/35 (34.3%) horses, bifid ribs in 3/35 (8.6%) horses, and only 4/35 (11.4%) horses had a normal length of the first rib on both sides. There was a moderately to highly significant association between the grades of left and right malformations of C6 and C7 and first ribs as well as between C6 and C7. A large number of malformations were visualized radiographically at the C-T junction using the newly described methods of latero-lateral and oblique radiographic projections, which allows for these features to be identified in living horses.

## 1. Introduction

In recent years, it has been recognized that malformations in the equine cervicothoracic (C-T) junction are present in many horse breeds and may be associated with a clinical presentation of local pain and restriction of motion, proprioceptive deficits, ataxia, lameness, postural abnormalities and unusual, even dangerous behaviors. Within the past twenty years, this syndrome has been variously described as equine caudal cervical morphologic variations (ECCMVs) [1], abnormalities of the ventral lamina of the sixth cervical vertebra (AVL-C6) [2,3,4], and equine complex vertebral malformations (ECVMs) [5,6,7]. The latter encompasses the myriad of osseous, ligamentous, vascular, nervous and muscle changes that can affect the C-T junction and the function of the brachial plexus [5,6,7]. Pathology of the cervical spine and the C-T junction can be associated with neck pain, reduced cervical range of motion, proprioceptive deficits, ataxia, forelimb and hind limb lameness, a base-wide forelimb stance, and abnormal behavior [2,5,8]. Advanced diagnostic imaging techniques for diagnosis of disorders of the cervical spine are available [9,10,11,12,13,14,15,16], but radiographic examination remains the first diagnostic imaging technique of choice to investigate the cervical vertebrae and the C-T junction due to practical reasons such as availability, noninvasiveness and standardizable features [17,18]. Radiological interpretation of this difficult area depends on the knowledge of the radiologist or veterinarian and the quality of the images [17,18,19]. Previous reports focused on C6 only to provide retrospective research about the anatomical variations versus malformations of C6 and C7 [3,4,20,21]. A previous study stated that it was difficult to image C7 and especially the first thoracic vertebra (T1) [6]. To the authors’ knowledge, the radiographic methods to image the first (R1) and second (R2) ribs using portable radiological devices in the field has not been described before. The aims of this study were to describe new radiographic methods to separately image the left and right side of the C-T junction, R1 and R2 and to describe the radiographic appearance of the malformations of C6, C7, T1 and the first and second ribs using a new detailed classification system. The authors hypothesized that the presented radiographic methods allowed for the identification of different grades of malformations of C6, C7, T1, R1 and R2.

## 2. Materials and Methods

### 2.1. Ethical Statement

This study was approved by the Institutional Review Board of the University of Veterinary Medicine Hannover (Foundation) and the state veterinary office from Lower Saxony, Oldenburg, Germany (Institutional Animal Care and Use Committee, IACUC) (33.19-42502-05-16A023) on 6 January 2017. The handling of the horses followed European Union guidelines for animal care and handling and the Guidelines of Good Veterinary Practices.

### 2.2. Study Population and History

Radiographs of the C-T junction from C6 to T2 were obtained from 39 warmblood horses from January 2021 to August 2022, due to requests by owners for clinical evaluation. There were 22 females, 14 intact males and 3 geldings, ranging in age from 6 months to 18 years with a median age of 5 years. All were warmblood horses including 14 Westphalian (Westphalian Breeders’ Association, Münster, Germany), 8 KWPN (Royal Warmblood Studbook of the Netherlands, Ermelo, The Netherlands), 6 NRPS (The Netherlands Riding Horse and Pony Studbook, Lunteren, The Netherlands), 4 AES (Anglo European Studbook, Schijndel, The Netherlands), 3 Oldenburg and 1 Belgian and Hanoverian warmblood, Trakehner and Zangersheide.

### 2.3. Radiographic Method

Radiographic images of the caudal cervical column including the C-T junction were obtained in the standing horse. All images were acquired using a DEVO-II digital radiographic (DR) system (Fuji, Japan) with generators capable of 90 to 200 kV and 20 to 30 mA and veterinary image management software (Equine Veterinary solutions, vsol 6), with the same operators performing all the procedures.

**Figure 1:**
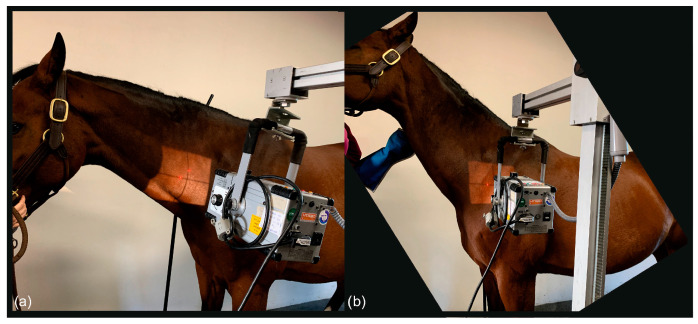
(**a**) The photo shows the position of the neck to obtain a latero-lateral radiographic projection of C6-C7; the horse stands with all legs squared and the head in a neutral position. (**b**) The photo shows the position of the neck to obtain a straight latero-lateral radiographic projection from C7 to T2, with the horse standing with all legs squared and the head manually raised with the nose at withers height (body weight of the horse: 530 kg).

Exposure factors varied, depending on the size of the horse, ranging from 72 to 78 kV and 2 to 2.5 mAs for the caudal cervical column and 74 to 84 kV and 3.4 mAs for the cranial thoracic vertebrae and the first and second ribs. Each horse was positioned with all legs square and perpendicular to the ground for the lateral and oblique images of the C-T junction from C6 to T2. The head and neck were held in a neutral position, the neck being horizontal, or elevated manually by an operator up to 45 degrees with the horse’s nose at or above the withers level, as shown in Figure 1.

To obtain latero-lateral radiographs of C7 to T2 in horses, the head was carefully elevated to raise the caudal cervical and cranial thoracic vertebrae, which are cranioproximal to the shoulder joint, and to avoid superimposition with the scapula (Figure 1).

**Figure 2:**
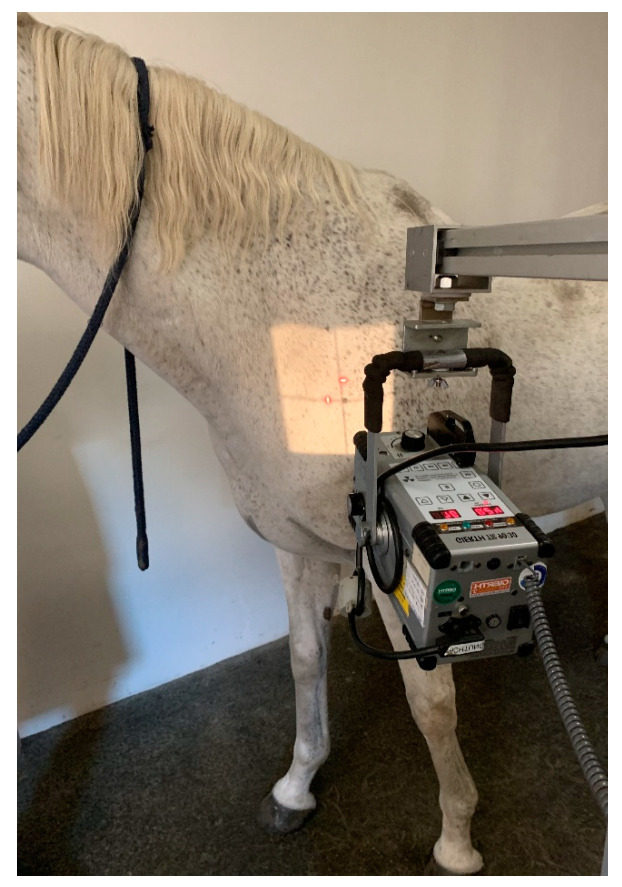
Photo showing the position of the neck to obtain the oblique radiographic projection of the right first rib. The left forelimb is positioned caudally, and the head is slightly elevated (body weight of the horse 580 kg).

For the projections of the first and second ribs, the forelimbs of the patient were positioned behind the vertical line. In some cases, only the forelimb close to the generator was positioned behind the vertical line and the contralateral forelimb maintained in a square position; the head was again, in some cases, elevated to obtain better images, as shown in Figure 2.

**Figure 3:**
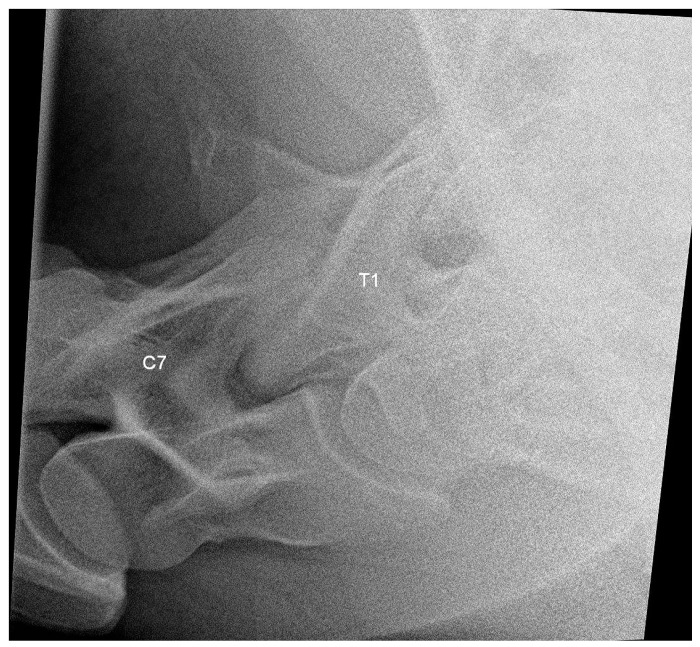
Latero-lateral radiographic image of C7 and T1 of a 6-year-old warmblood mare with a normally shaped C7 and T1, obtained with a small detector plate measuring 26 by 33 cm. The head of the horse is to the left.

The latero-lateral projections of C6, C7, T1 and T2 were acquired in all horses at a focus-to-film distance of 80 cm using a suspended vertical cassette holder and tripod. For the acquisition of the latero-lateral projections of the C-T junction from C6 to T2, the X-ray beam was centered on the vertebral canal perpendicular to the center of the vertebra and parallel to the ground. The main landmark for interpretation was the identification of C7 and T1 on the radiograph (Figure 3), which allowed for the correct identification of C6 and C7.

**Figure 4:**
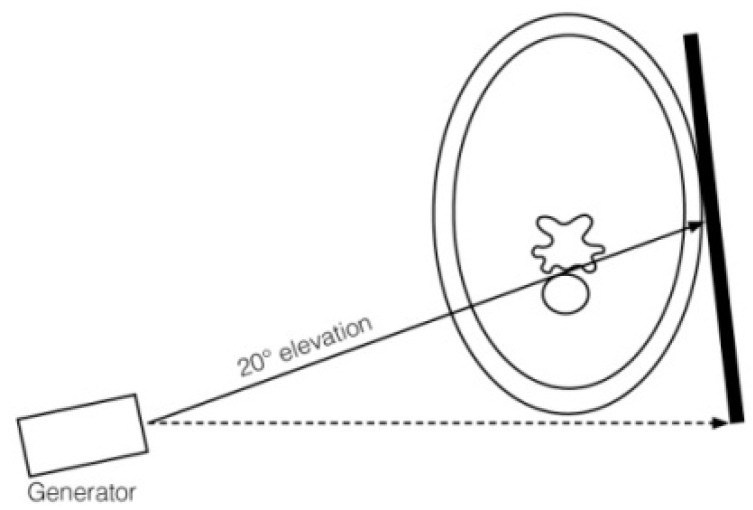
Drawing of generator, plate position and orientation to obtain a 20° ventrodorsal projection to image the ventral laminae of C6 and C7. The dashed line represents the horizontal line that intersects the underside of the neck as a tangent, and the arrow indicates the viewing direction in which the horizontal line is aligned by the examiner. The arrow on the solid line indicates the direction of the X-ray beam.

**Figure 5:**
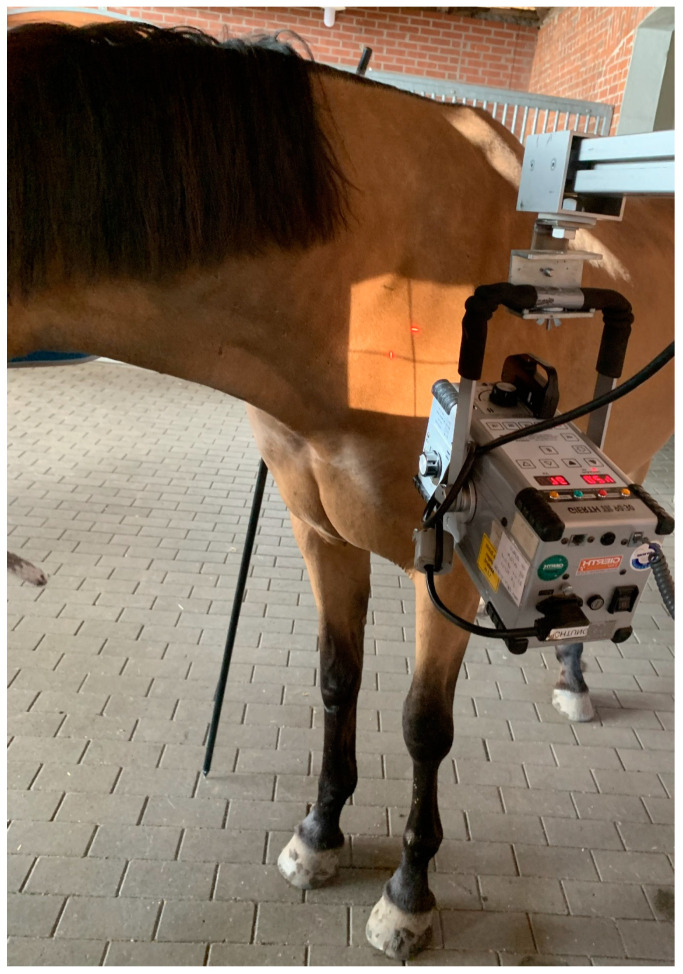
Photo of a horse with the neck in neutral position to obtain the radiographic projection of the ventral aspect of the right C7 and the right-ventral lamina of C6 (body weight of the horse 565 kg).

Oblique projections were performed from the right as a 20° to 45° right-ventral to left-dorsal oblique projection and from the left as a 20° to 45° left-ventral to right-dorsal oblique projection, both shown in Figure 4, and the X-ray beam was centered on the ventral side of the vertebral bodies (Figure 4 and Figure 5).

**Figure 6:**
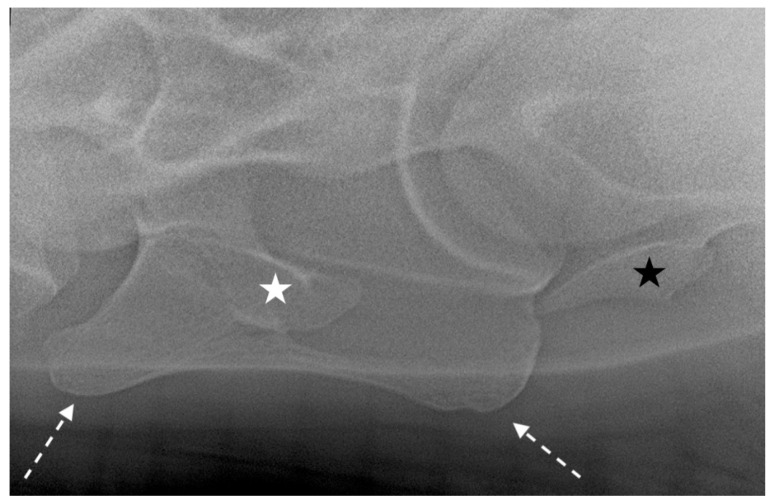
Left 25° left-ventral to right-dorsal oblique projection in a 10-year-old warmblood mare. The right-ventral lamina of C6 (white dotted arrows) is clearly visible and superimposed on the transverse process (white star). The right transverse process of C7 is also visible (black star). The head of the horse is to the left.

A 20° to 45° ventrodorsal oblique radiographic projection allowed for the identification of the ventral laminae of C6 and, in the case of transposition, also of C7, as shown in Figure 6. 

**Figure 7:**
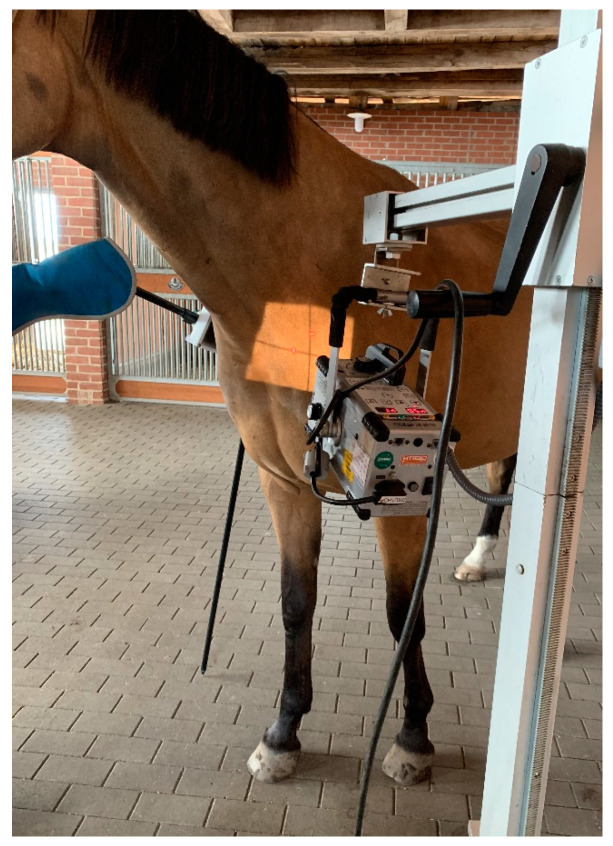
Photo of a horse positioned for radiography of the first right rib. The left forelimb is positioned caudally. The X-ray beam is centered slightly dorsocaudal to the supraglenoid tubercle of the shoulder closer to the generator (body weight of the horse 565 kg).

Oblique radiographic projections for the evaluation of the first and second ribs were obtained with lateral 10° cranial 10° ventro-caudodorsal oblique views. This projection was performed from the right side as a right 10° cranial 10° ventral-left caudodorsal oblique view and from the left side as a left 10° cranial 10° ventral-right caudodorsal oblique view to image the left and right ribs, respectively. The focus was on the supraspinatus muscle, 10 to 15 cm proximal to the supraglenoid tubercle of the scapula, which was closest to the generator (Figure 7).

**Figure 8:**
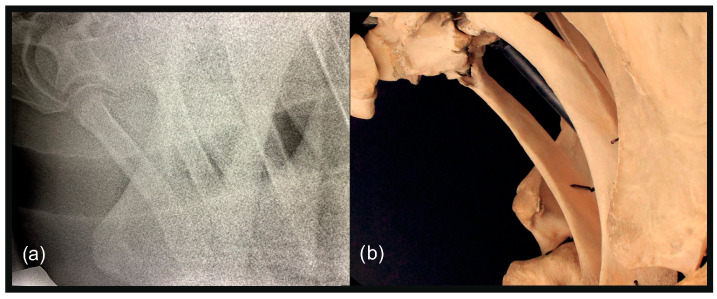
(**a**) Left 20° cranial 10° ventral-left caudodorsal oblique projection showing the right first and second ribs separately. The head of the horse is to the left. (**b**) Photo of the anatomical structures as projected in (**a**).

To image T1 with the head of the first rib, the focus-to-film distance was reduced to as close as 60 cm to minimize radiation exposure and maximize image quality. The cassette was installed in a suspended vertical cassette holder and positioned as close to the neck and shoulder as possible, as shown in Figure 5, Figure 6 and Figure 7, to minimize distortion on the radiographs (Figure 8).

### 2.4. Classification System

**Figure 9:**
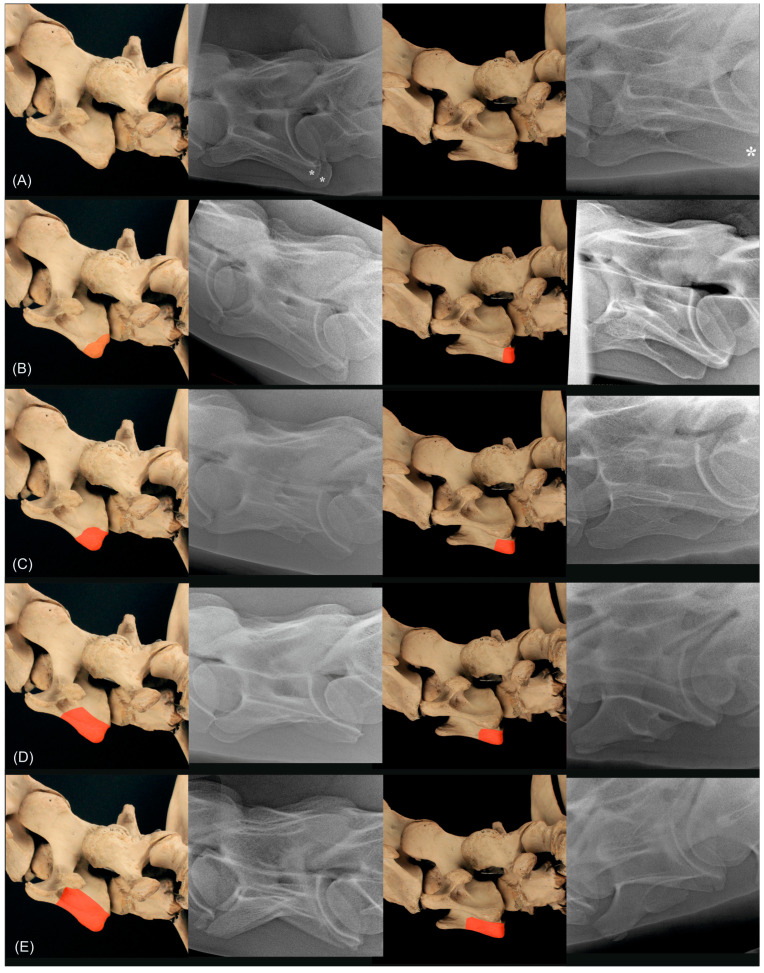
Anatomical specimen with the normal C6 cervical vertebra (**A**) and schematic representations of the four different grades marked in red ((**B**) corresponds to grade 1, (**C**) corresponds to grade 2, (**D**) corresponds to grade 3, (**E**) corresponds to grade 4) of the classification system for C6, in the first column from left lateral (90°) and in the third column from 20° to 45° left-ventral to right-dorsal oblique. In the second and fourth column are radiographs of different horses showing a normal C6 (**A**), a C6 grade 1 (**B**), a C6 grade 2 (**C**), a C6 grade 3 (**D**) and a C6 grade 4 (**E**) in the left latero-lateral projection (second column) and 20° to 45° left-ventral to right-dorsal oblique projection. The asterisks ((**A**), second and fourth column) mark the caudal quarter of the ventral lamina of C6.

**Figure 10:**
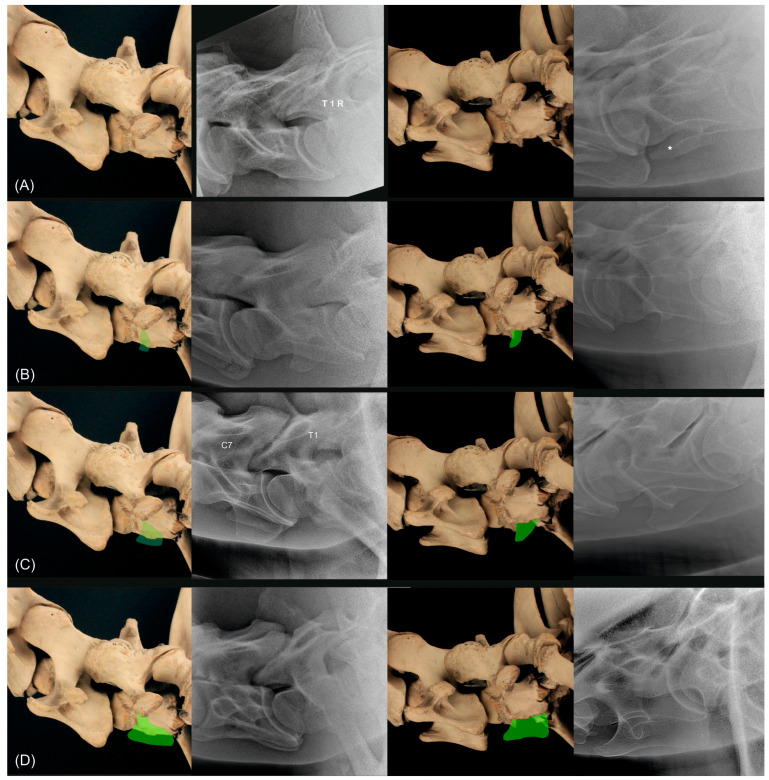
Anatomical specimen with the normal C7 cervical vertebra (**A**) and schematic representations of the three different grades indicated in green ((**B**) corresponds to grade 1, (**C**) corresponds to grade 2, (**D**) corresponds to grade 3 of the classification system for C7), in the first column from left lateral (90°) and in the third column from 20° to 45° left-ventral to right-dorsal oblique. In the second and fourth column are radiographs of different horses showing a normal C7 (**A**), a C7 grade 1 (**B**), a C7 grade 2 (**C**) and a C7 grade 3 (**D**) in the left latero-lateral projection (second column) and 20° to 45° left-ventral to right-dorsal oblique projection. The asterisk in the radiograph ((**A**) in fourth column) marks the transverse process of C7. T1 and right side R are marked in the radiograph ((**A**), second column) and C7 and T1 are marked in the radiograph ((**C**), second column).

**Figure 11:**
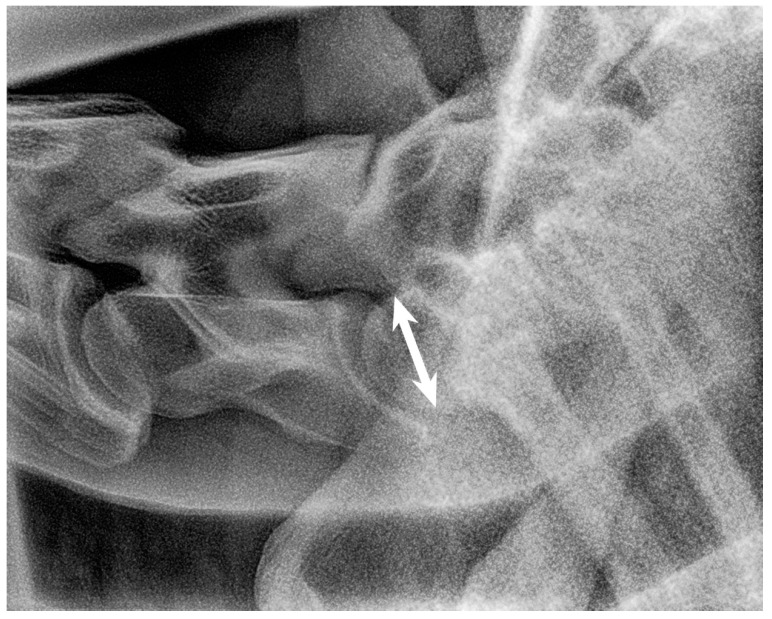
Left latero-lateral radiographic projection showing the first and second ribs, obtained in a 3-year-old warmblood mare. C7 has a normal shape, as does T1 with first ribs and T2 with second ribs. The spinous process of C7 is not clearly visible, most likely because it is absent. The spinous process of T1 has medium height but is not completely visible. White arrow shows the measurement of the height of the cranial aspect of the vertebral body of T1 used as a reference for the grading classification. Head of the horse is to the left. Note that the superimposed first ribs are wider than the second ribs, reflecting the normal shape of these ribs.

**Figure 12:**
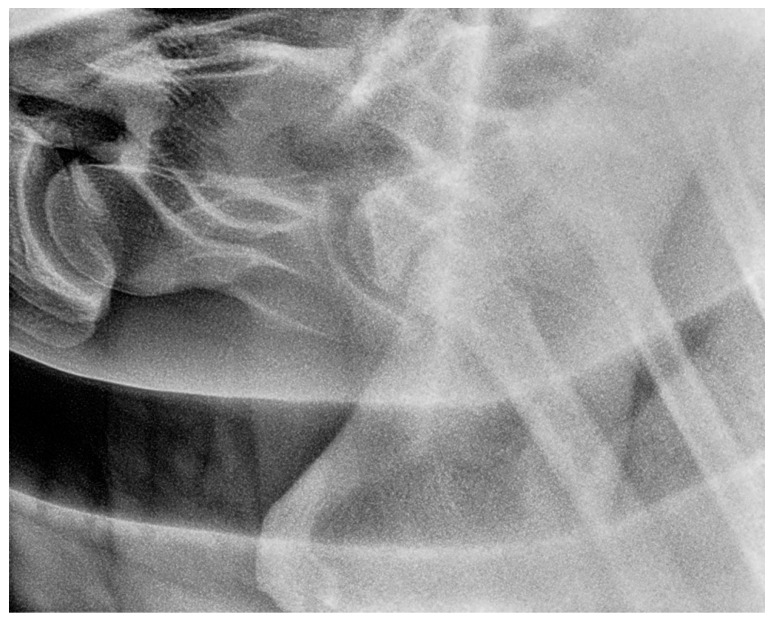
Left 5° cranial 15° ventral right caudodorsal oblique radiographic projection showing the right first and second ribs, obtained in a 3-year-old warmblood mare (the same horse as in Figure 11). The head of the horse is to the left.

**Figure 13:**
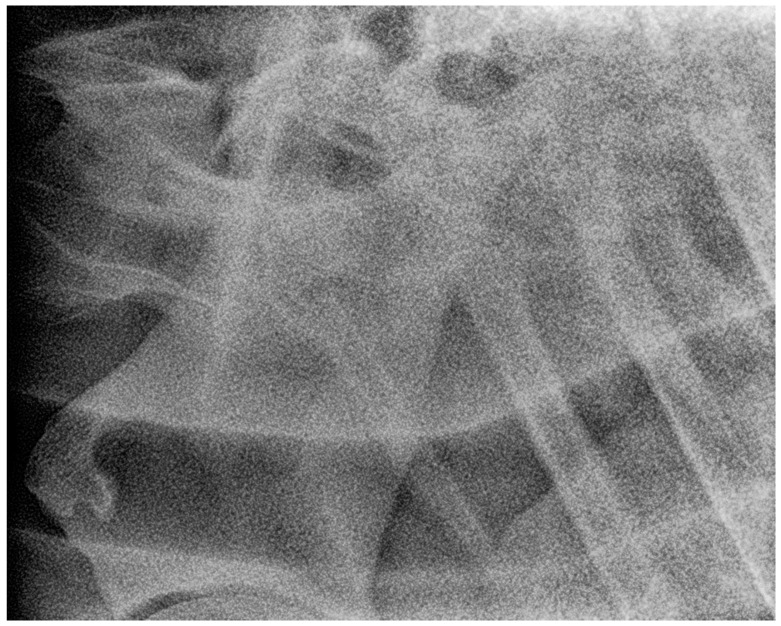
Right 10° cranial 20° ventral-left caudodorsal oblique projection showing the left first and second ribs, obtained in a 3-year-old warmblood mare (the same horse as in Figure 11 and Figure 12). The first left rib is large rudimentary (Grade 3/1). The head of the horse is to the left. Second rib is grade 1.

**Figure 14:**
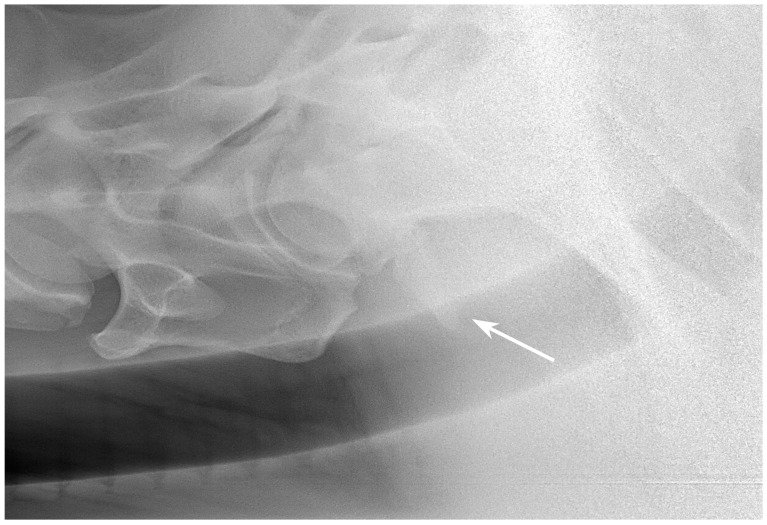
Left 10° cranial 25° ventral-right caudodorsal oblique projection showing the right first and second ribs, obtained in a 4-year-old warmblood gelding. A rudimentary first rib is visible (arrow) (Grade 3/3) and C7 shows complete transposition of the ventral lamina (Grade 3). Second rib width is classified with grade 1. The head of the horse is to the left.

All radiographs were evaluated by four trained analysts, all with more than 10 years of experience in the interpretation of radiographic images of the equine neck. Before image analysis, 20 sets of radiographs were selected randomly and evaluated by the four experienced operators to achieve precision (>99%) in interpretation. After analysis of all images, different classification systems were developed to describe the shape of C6, as demonstrated in Table 1 and Figure 9; C7, as demonstrated in Table 2 and Figure 10; and the first and second ribs, as demonstrated in Table 3, Table 4 and Table 5 and Figure 11, Figure 12, Figure 13 and Figure 14; the detection of a bifid first rib was recorded. All malformations were recorded as right-sided, left-sided or bilateral.

### 2.5. Data Analysis

For statistical analysis, data from the geldings and intact males were pooled as males. Data were reported as frequencies and percentages. The likelihood ratio chi-square (LR chi square) or Fisher’s exact test was used, when appropriate, to test for differences between the radiographic findings and sex. Calculation of Cramer’s V coefficient was used to test for associations between grades of malformations between the left and right sides of the same vertebra and between malformations in different anatomical structures. These statistical tests were performed using SAS, version 9.4 (Statistical Analysis System, Cary, NC, USA, 2023). Significance was set at *p*-value < 0.05.

In addition, we employed a multivariate generalized mixed model to estimate variances and covariances among grades of C6, C7, length and width of the first ribs and width of the second ribs. Fixed effects tested were sex, breed, age class, body side and anatomical structure (C6, C7, first rib length, first rib width, second rib width). Horses under study were treated as a randomly distributed effect. The final model only included the effects of the horse, body side and type of trait. Body side and type of anatomical structure were significant at *p*-values of 0.0141 and <0.0001. All other effects and all two-interactions among the fixed effects were not significant and thus, omitted in the final model.

Y_ijkl_ = µ + body side_i_ + anatomical structure_j_ + horse_k_ + e_ijkl_,

where Y_ijkl_ = grade of the respective anatomical structure and e_ijkl_ = residual error.

Statistical analyses for this model were performed with SAS, version 9.4 (Statistical Analysis System, Cary, NC, USA, 2023) using the procedure MIXED for a correlated random coefficient model. This model allowed us to estimate variances and covariances for the grades of the different anatomical structures as well as the correlations among the grades for C6, C7, first rib length, first and second rib width.

## 3. Results

### 3.1. Study Population

The study population comprised 39 warmblood horses, of which 17 were male and 22 female. The horses came from three countries, including 21 German, 16 Dutch and 2 Belgian warmblood horses. Mean age of horses studied was 6.63 ± 5.06 years with first and third quantiles of 2 and 11 years, respectively. The age of the horses was regarded in three classes with the distinction between up to 5 years old (*n* = 13), 6–10 years old (*n* = 13) and older than 10 years (*n* = 13). Sex by country of origin deviated not significantly from an equal distribution (*p*-value = 0.2). Age by sex and age by country of origin were evenly distributed (*p*-value = 0.2 and 0.7). Thirty of thirty-nine warmblood horses had radiographic images suitable for evaluation of C6 to T2 including the first and second ribs (Table 6). In 4/39 horses, the second rib pair was not imaged with sufficient quality. In further 2/39 horses, both left ribs could not be evaluated because of poor image quality and the heel effect. In the remaining 3/39 horses, either the right or left first rib or both second ribs and the left first rib could not be imaged due to the caliber of the horses. There were no significant effects of sex, age and country of origin (Belgium, Germany, The Netherlands) on malformations of C6, C7, the first rib and second rib using LR chi square tests (*p*-values > 0.08–0.9).

### 3.2. Distribution of Malformations

The C6 was classified as normally shaped in 11/39 and 3/39 horses on the right and left side, respectively (Table 7). In 28/39 and 34/39 horses, the ventral lamina of the C6 was reduced to less than 25% (grade 4) on the right and left side, respectively. More horses with a reduction in ventral lamina were observed on the left side. In 27/39 horses, there was almost complete aplasia of the ventral lamina (grade 4) on both sides. In all other grades, each one horse showed malformations on the left side only. The correlation between the body sides via Cramer’s V was 0.460 and significant (*p*-value = 0.046).

When we classified the horses with grades 1–4 as affected either on the left or right side, 27/39 horses showed bilateral malformations on C6, and only 2/39 horses were normal on both sides. The correlation between body sides via Cramer’s V was 0.247, and there was no significant correlation between body sides (*p*-value = 0.148).

C7 was normal in shape in 11/39 and 3/39 horses on the right and left sides, respectively, and the remainder showed transpositions of the ventral lamina from C6 to the ventral side of C7 (Table 7). In 8/39 and 13/39 horses, the transposition was bilateral with grade 2 and 3, respectively. The correlation between the body sides via Cramer’s V was 0.4 and significant (*p*-value = 0.01).

The differentiation between normal and horses with malformations of C7 resulted in 2/39 and 27/39 horses with bilaterally normal and bilaterally malformed C7, respectively. The correlation between body sides via Cramer’s V was 0.2, and there was no significant correlation between body sides (*p*-Value = 0.15).

Cramer’s V between the grades within the right and left side for the malformations of C6 and C7 was 0.607 (*p*-value < 0.0001) and 0.9 (*p*-value < 0.0001), respectively. In 14/39 (35.9%) and 22/39 (56.4%) horses, there was the highest grade of abnormality affecting the right and left sides in C6 and C7, respectively. All other horses had different grades of severity affecting the left and right side. Joint distributions of malformations of C6 and C7 are provided in Supplemental Tables S1 and S2. Comparing contralateral sides indicated fewer close associations (*p*-value = 0.4 and 0.37, when comparing right C6 with left C7 and left C6 with right C7, respectively) than for comparisons within the same side.

Comparison of malformations between C6 and C7, when horses were classified as normal and affected for grades encoding malformations provided significant correlations for each side (Table 8). Most horses had malformations on both vertebrae, C6 and C7 (37/39, 94.9%).

The most common combinations of concomitant malformations of C6 and C7 were bilateral malformations of grade 4 in C6 and bilateral malformations of grade 3 in C7 (*n* = 13; 33.3%), followed by bilateral malformations of grade 4 in C6 and bilateral malformations of grade 2 in C7 (*n* = 8; 20.5%). Unilateral grade 4 malformations of C6 and grade 3 malformations of C7 were next frequently seen in four (10.3%) horses.

Consistent findings among the length of the right and left first rib were found in 10/35, 2/35 and 4/35 for grade 3 and 4 and normal appearance, respectively (Table 9). In two horses, an abnormal length was detected affecting both sides, including the cases of missing ribs.

In 3/35 (11.4%) horses, one or both first ribs were missing, with two (5.7%) horses having both first ribs missing and one horse (2.9%) having the left first rib missing.

Four (11.2%) horses showed a normal length of the first rib bilaterally, but twelve horses (39.5%) had a normal length of the first rib only on one side, including the right rib of eight horses and the left rib of four horses.

There was no significant association between grades of malformations in the length between the left and right side of the first rib, including missing and bifid ribs (Cramer’s V = 0.491; *p*-value = 0.194).

Grade 3 was the most common finding for the width of the first ribs with 13/37 (35.1%) on both sides, 18/37 (48.7%) on the right side, and 23/37 (62.2%) on the left side (Table 9). Normal width of the first ribs was found either in 10/37 (27.0%) on the right side or in 9/37 (23.7%) on the left side. There was no horse with normal width of the first rib pair.

Distributions of malformations of the length and width of the first ribs by body sides are provided in Supplemental Tables S3 and S4.

All combinations of concomitant malformations of C6, C7 and the first ribs were found (Table 10). Most commonly were malformations affecting C6, C7 and the first ribs. In cases of missing first rib (*n* = 3), unilaterally or bilaterally, these horses had malformations of grade 4 for C6, but malformations of grade 2 or 3 for C7.

The most common malformations of C6, C7 and the length of first ribs were bilateral malformations of grade 4 in C6, bilateral malformations of grade 3 in C7 and bilateral grade 3 of the first ribs. All other combinations were found in different combinations in single or two cases.

The second rib was graded as either normal, grade 1 (wide) or grade 2 (very wide). In 7/31 (22.6%) horses, the left second rib was graded as normal, in 19/31 and 4/31 horses with grade 1 and 2, respectively (Table 11). For the right second rib, we found 12/34 (35.2%) normal horses, 18/34 horses (52.9%) with grade 1 and 2/34 (5.9%) horses with grade 2. Malformations of the second pair of ribs were most common in horses with malformations of C6, C7 and the first ribs.

Distributions of malformations of the width of the second ribs by body side as well as of the length and width of the first ribs by body side with the width of the second ribs are provided in Supplemental Tables S5–S7.

Horses in this study showed significant estimates for the proportion of the variance estimated for C6 and the first rib length (Table 12). All correlations were positive. Significant correlations were found for grades between C6 and C7 as well as between length of first rib and C6 and C7. First and second rib width as well as first rib length and second rib width were highly significantly correlated.

## 4. Discussion

In this study, we describe a protocol for a new field radiographic method to investigate the presence of malformations at C6, C7, the first (R1) and second ribs (R2). In addition, we developed a new classification system to describe the malformations detected in caudal cervical vertebrae and the first and second ribs. Compared with previous studies [2,3,4,21], latero-lateral and oblique images of the caudal cervical vertebrae were obtained to highlight the ventral side of C6 and C7 bodies. Therefore, the focus of the X-ray beam was directly on this area of interest. A method of imaging the ventral laminae of C6 using oblique projections has been described previously, and the possibility of projecting each lamina of C6 individually has been demonstrated [1], but in the present study, a ventrodorsal instead of a dorsoventral orientation of the X-ray beam was chosen because it was easier to perform in non-sedated horses. We rarely sedated patients for the procedure to X-ray C6 to T2. Unsedated horses stand more straight and stable. Sedated horses tend to lean to one side, move more unexpectedly, react more on flies and usually drop the thorax more ventrally. This way, it is harder to avoid superimposition of T1, T2, R1 and R2 with the scapula. Since the horse is a flight animal, non-sedated horses may become nervous when the generator is lifted on the stative high above the neck of the horse. If the horse unexpectedly moves its head close to the high-positioned X-ray generator, this can lead to a dangerous situation for the equipment, the handler and the horse itself. This situation did not happen in this study population because the authors took time to make the horses get used to the equipment and the procedure. Also, the low positioned the X-ray generator was well tolerated by all horses in this study. The 20° ventrodorsal orientation of the X-ray beam also avoided superimposition of the transverse process of C7 with a potential transposition of the ventral lamina, which can happen with a 45° ventrodorsal orientation [1], thus obscuring the transposition. In accordance with the initial hypothesis, diagnostic radiographic images, which allowed for evaluation of the malformations of C6, C7, R1 and R2, were obtained in all but four horses. In three horses on the left side and in one horse on the right side, R1 and R2 were not imaged mainly due to the caliber and instability of these horses. The reason for not succeeding to image R1 and R2 was mainly the width of the horses in the shoulder region and the position of the thorax. These four horses presented themselves with a ventrally dropped thorax and due to body instability, lifting of the head and neck was restricted.

**Figure 15:**
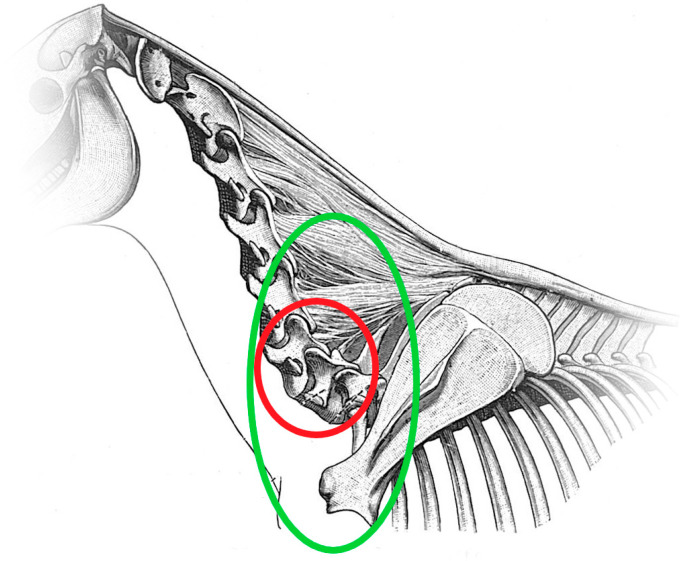
Diagram of the horse neck and cranial thorax, viewed from the left side. The red circle indicates the area of C6 and C7. The green circle highlights the whole region of C6, C7, T1, T2, R1 and R2 that this study focuses on.

The radiographic method described also allowed for evaluation of the second ribs in a limited number of cases. The mobile X-ray generators did not have sufficient power to penetrate the most caudal portion of the C-T junction, especially in very large and massive horses with wide shoulder-to-shoulder distance, as shown in Figure 15. Individual changes in the alignment of the scapula and its overlap with the second ribs may also interfere with the acquisition of good images of this area.

**Figure 16:**
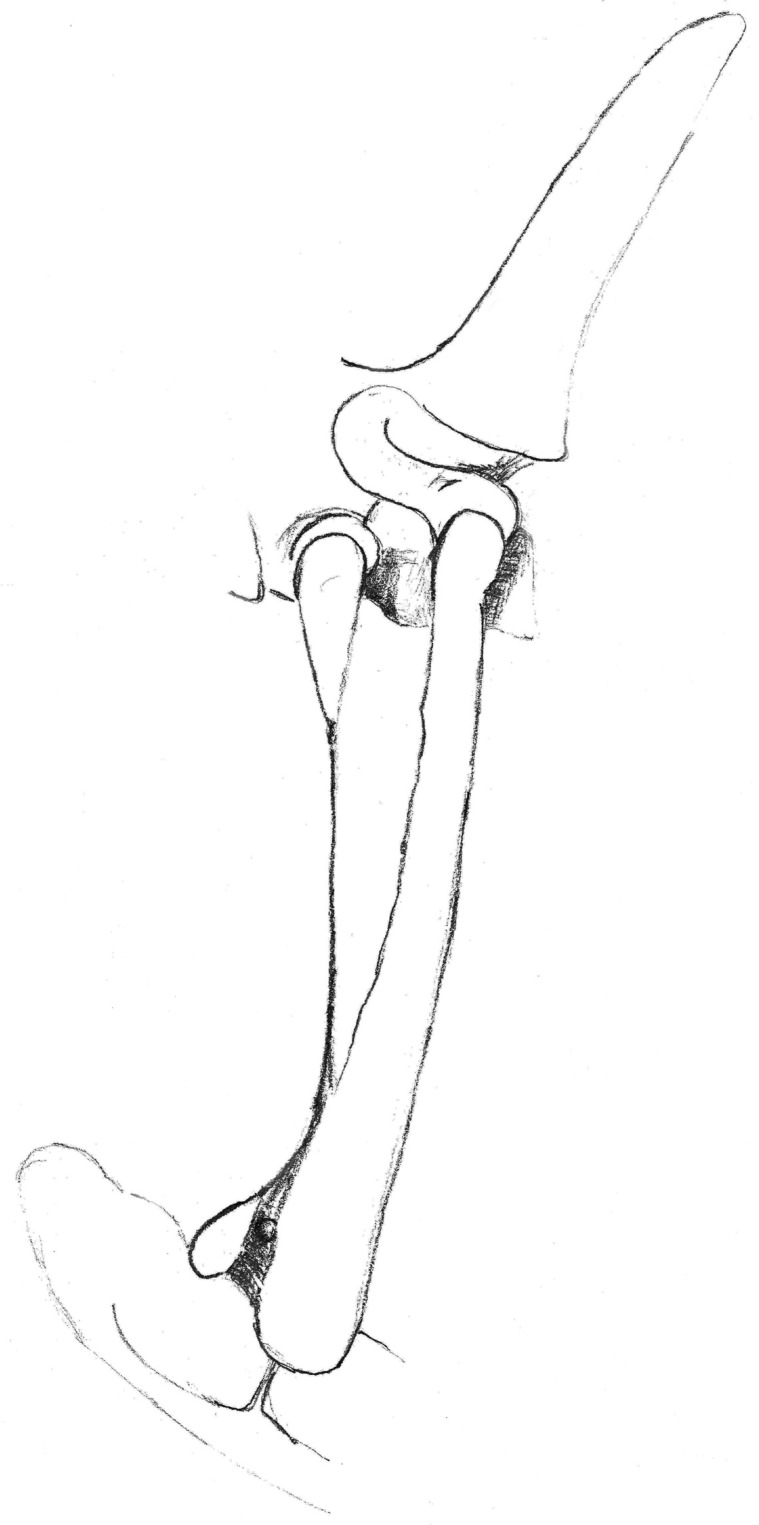
Drawing of a left malformed rudimentary first rib and malformed second rib. View from the left side.

**Figure 17:**
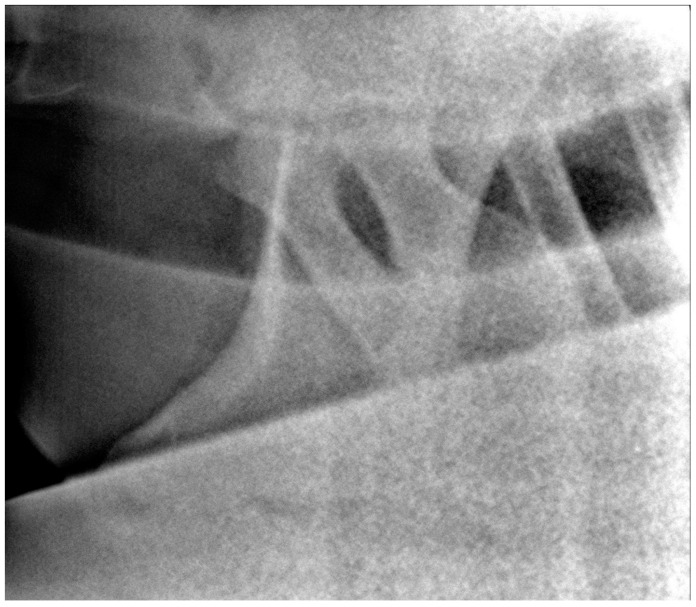
Left 10° cranial 25° ventral-right caudodorsal oblique projection showing fused right first and second ribs (bifid rib), obtained in a 1-year-old warmblood mare. The head of the horse is to the left.

**Figure 18:**
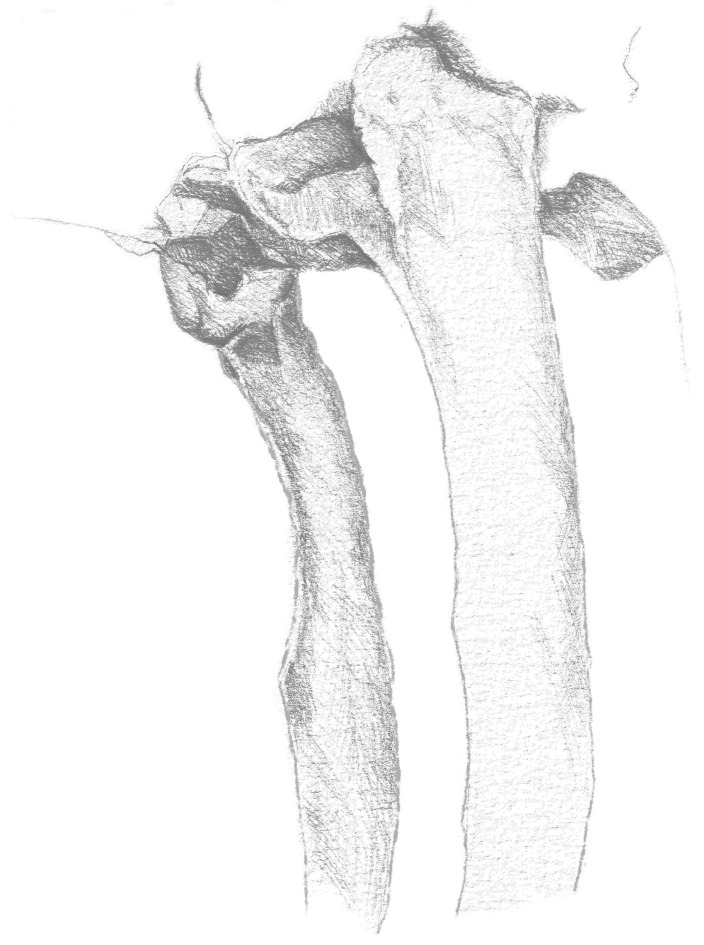
Drawing of the first thoracic ribs, showing a normal shape. Slightly cranial view from the left side.

Previously, malformations of this region were described based on data obtained by dissections [5,6,7,22,23], by radiographic examinations using nonportable X-ray generators [2,3,4,21] or using computed tomography [20]. Previous studies focused mainly on malformations of C6 and C7, recognized on latero-lateral radiographic projections of the C-T junction and transposition of the ventral lamina of C6 onto the ventral aspect of C7 [1,2,3,4,21,24]. According to our experience, correct oblique views of C6 and C7 are very important. On latero-lateral radiographs, it is possible that unilateral aplasia of one of the ventral laminae of C6 may be underdiagnosed because of superimposition of the normal laminae, especially when radiographic quality is poor. Diagnosis of congenital malformations of C6 and C7 is difficult only on latero-lateral images. For these reasons, oblique radiographs should be used in future prevalence studies to avoid misinterpretation. We detected a large number of horses with malformations of C6 and C7, probably due to the use of oblique projections [2,3,4,6,20,21]. On an X-ray set of the lower neck only, that shows a complete bilateral aplasia of the ventral laminae of C6 (grade 4), the presentation of C6 can be easily misinterpreted as C5, and when there is complete bilateral aplasia of the ventral laminae of C6 (grade 4) and complete transposition of the ventral laminae to the ventral aspect of C7 (grade 3), C7 can be mistaken for C6 [1]. With complete bilateral aplasia of the ventral laminae of C6 (grade 4), complete transposition of the ventral laminae to the ventral aspect of C7 (grade 3) and bilateral complete aplasia of the first sternal ribs, T1 may almost look like C7, and in this hypothetical case of “perfect” congenital malformations of C6, C7 and T1, they could appear as C5, C6 and C7, respectively. This special case would not be mistaken if the cervical vertebral column would have been imaged with the use of markers onto C5 and vertebrae were counted from cranial to caudal, up to T1. These horses have been judged in the past as normal and not affected with congenital malformations of C6 and C7 on radiographs, but functionally, they have eight cervical vertebrae. This condition was found in two cases in this study, and only the correct identification of T1, through identification of C7 and T2 with the second ribs, allowed for the correct interpretation of the malformations. An experienced veterinarian should be able to distinguish a C6 with bilateral grade 4 aplasia of the ventral laminae from a normal C5. At C3 to C5, a marked ventral crista with the caudal tuberculum is present, but not at C6 and C7. The lateral processes of C3 to C5 measured from the most cranial aspect of the ventral tubercle to the most caudal aspect of the dorsal tubercle are getting shorter from C3 to C5. Often, C6 shows a triangular shape of the dorsal lamina of the vertebral arch. The most caudal extension of the dorsal lamina of the vertebral arch (roof of the spinal canal) of C6 is in general shorter than the floor of the spinal canal. For C5, the opposite is the case, as the roof of the spinal canal reaches far more caudally than the floor of the spinal canal. It is well known that the dorsal spinous processes of C7, T1 and T2 have a specific shape [19], and the authors agree with previous studies showing that in cases of transposition of the lamina from C6 onto the ventral aspect of C7, the dorsal spinous processes of C7 and T1 were reduced and altered in shape and size [4], making interpretation of radiographs difficult. It is very important to be able to accurately identify the presence or absence of malformations of C6, C7, R1 and R2 to help breeders decide how to manage horses with this condition. In the authors’ experience, a similar situation can also occur during the prepurchase examination when the caudal cervical vertebrae are imaged only with latero-lateral projections without adequate visualization of the C-T junction, including T1. Based on the data from this study, we suggest that diagnosis of the C-T junction can only be made if C6, C7, T1 and at least the cranial half of T2 are imaged with the first and possibly the second ribs in latero-lateral and oblique projections. T1 and T2 have a unique shape due to the shape of the spinous processes and ribs [4]. To the authors’ knowledge, there are no other studies that focus on imaging the ventral aspect of the vertebral bodies of T1, T2 and of R1 and R2. The use of oblique images and correct identification of T1 can help practitioners correctly identify malformations of the C-T junction, avoiding the errors previously reported [1]. Also, the images aid in grading the extent of malformations of the C-T junction. In the authors’ experience, grading the malformations of the C-T junction is important because some malformations may be responsible for cervical-related clinical signs, and identification may help to understand the implications of malformations of the C-T junction for each individual horse [7]. The lack of grading assessment may explain the conflicting results reported in the literature on the clinical significance of congenital malformations of C6 and C7 [2,3,5,20]. Recently, a grading system for the reduction in the cranial and caudal ventral tubercle of the ventral transverse process on C6 was reported [23]. The grading system for the caudal ventral tubercle is similar to our system for the shape of the ventral lamina of C6 based on radiographs. While the system published by May-Davis et al. (2023) was developed for the ventral view of osseous specimens [23], our system is designed for the assessment of radiographic images and is therefore suitable for use in clinical medicine. ECRM includes malformations defined as aplasia, hypoplasia, synostosis, malformations and pathological malformations of R1 and R2 [5,22,25]. A previous study reported six cases of bifid ribs, also called first and second rib synostosis, describing the fusion of R1 and R2, as shown in Figure 16 and Figure 17, and compared to normal R1 and R2 (Figure 18). The abnormality was considered the possible cause of forelimb lameness in four horses [25]. In 3/39 horses in this study, unilateral bifid ribs were detected, and in one case, this was considered the possible cause of cervical pain. In the present study, in 17/33 (51.5%) and 21/31 (67.7%) horses on the right and left side, respectively, ECRM and congenital malformations of C6 and C7 were concomitantly recognized. ECRM seems to be relatively common in horses with congenital malformations of C6 and C7 and it may be considered as its caudal extension into the thoracic spine. However, one case (3-year-old warmblood mare shown in Figure 11, Figure 12 and Figure 13) showed an exception to this rule because C6 and C7 showed no malformations, but the ventral laminae of C6 appeared smaller compared to horses with a normal C6 and the left first rib was long and thin rudimentary. This highlights the importance to include images of the first and possibly the second ribs in horses that are affected with congenital malformations of C6 and C7 and horses with a normal appearance of C6 and C7. The prevalence of ECRM in horses without congenital malformations of C6 and C7 requires further study.

To image the first ribs, the positioning of the horse is crucial. It is important that T1 is positioned cranioproximally to the scapula because C7, T1 and T2, and also C6 in some horses, can be obscured by the scapula. In some cases, the best position is achieved by maintaining the horse with a neutral position of the neck, but in others, maximal lifting of the head or careful cranial traction applied to the rami of the mandible and lifting the head about 20–100 cm dorsally, compared to a neutral position, assisted in forward–upward extension of the C-T junction relative to the scapula. In exceptional cases with massive musculature, we suggest retracting the forelimb on the opposite side of the rib to be imaged. Caution is needed, because horses can experience pain, lean or stumble because retraction of the forelimb can destabilize horses with instability or pain of the C-T junction, especially if they were sedated. Sedated horses may also rotate in the thorax so that it becomes difficult to obtain the correct straight projections. Avoiding sedation during the X-ray procedure of the lower neck may help, because sedated horses can drop their thorax quite dramatically, so in this study, no sedation was administered to horses during the radiographic examination.

Several other findings in the C-T junction have been previously described by dissection, including the presence of two additional arterial foramina associated with transposition of the ventral lamina to C7, through which the vertebral arteries passed. This is not considered normal [2,5,7,26]. The possibility to identify their presence and possibly their radiographic appearance should be investigated.

It was not always easy to obtain good images of the first ribs on every horse for a number of reasons related to positioning. Some horses had different postures, leaning on one front leg only or standing with their front legs wide apart, and some were unable to tolerate the elevation of the head and neck due to pain in the lower neck. Most horses with missing ribs presented with a cranioventrally positioned thorax, making it easier to obtain radiographs. Horses with a caudodorsal position of the thorax make it almost impossible to image the first ribs without superimposing the scapula. In these cases, classification of both second ribs for malformations was not possible. The operators focused mainly on imaging the dorsal half of the first and second ribs, so it was possible that long rudimentary ribs were missed. In some cases, the superimposition of the scapula prevented the full shape of the first and second ribs from being visualized, despite changes in the positioning of the horse. Radiographs are only a two-dimensional representation of a three-dimensional structure, so many findings regarding the shape and possible asymmetry of C6 and C7 and the complete shape of the first and second ribs were not included in this study.

## 5. Conclusions

A wide range of malformations of the caudal cervical vertebrae and the C-T junction were detected using newly developed radiographic methods in warmblood horses. This study demonstrated that it is possible to image the proximal third or half of the first and second ribs and their junction with T1 and T2 in warmblood horses. In order to obtain comparable data, a classification system for malformations of C6, C7, first and second ribs was proposed. Correct identification of C6 and C7 on radiographs is indispensable to avoid misinterpretation in cases with bilateral aplasia of the ventral laminae of C6. Analysis of data from 39 warmblood horses revealed positive associations between all malformations of C6, C7, R1 and R2. Further prospective studies may use these radiographic methods and grading systems to evaluate the biomechanical, neurological and postural effects for horses with malformations of C6-T2, first and second sternal ribs and the associations between the presence of malformations of the C-T junction and clinical signs.

## Figures and Tables

**Table 1:** Classification system applied for the evaluation of the shape of the ventral laminae of C6.

Grade	Morphologic Appearance
Normal	Normal lamina
1	1–25% aplasia of the ventral lamina
2	26–50% aplasia of the ventral lamina
3	51–75% aplasia of the ventral lamina
4	76–100% aplasia of the ventral lamina

**Table 2:** Classification system applied for the evaluation of the shape of the ventral laminae transposed onto the ventral aspect of C7.

Grade	Morphologic Appearance
Normal	Absence of malformations (=no transposition)
1	<30% transposition
2	31–60% transposition
3	61–100% transposition

**Table 3:** Classification system applied for the evaluation of the width of the first rib.

Grade	Morphologic Appearance
Normal	75% of the height of the cranial aspect of the vertebral body of T1
1	74–51% of the height of the cranial aspect of the vertebral body of T1
2	50–30% of the height of the cranial aspect of the vertebral body of T1
3	<30% of the height of the cranial aspect of the vertebral body of T1
4	absent

**Table 4:** Classification system applied for the evaluation of the length of the first rib.

Grade	Morphologic Appearance
Normal	75% of the height of the cranial aspect of the body of T1
1—large rudimentary	74–51% of the height of the cranial aspect of the body of T1
2—medium rudimentary	50–30% of the height of the cranial aspect of the body of T1
3—small rudimentary	<30% of the height of the cranial aspect of the body of T1
4	absent

**Table 5:** Classification system applied for the evaluation of the width of the second rib.

Grade	Morphologic Appearance
Normal	30–50% of the height of the cranial aspect of the body of T1
1	51–75% of the height of the cranial aspect of the body of T1
2	76–100% of the height of the cranial aspect of the body of T1

**Table 6:** Survey on the distribution of successful X-rays in 39 warmblood horses.

Number	C6 Right/Left	C7 Right/Left	T1 Right/Left	First Rib Right/Left	Second Rib Right/Left
30	x/x	x/x	x/x	x/x	x/x
4	x/x	x/x	x/x	x/x	-/-
2	x/x	x/x	x/x	x/-	x/-
1	x/x	x/x	x/x	x/-	-/-
1	x/x	x/x	x/x	-/x	x/x
1	x/x	x/x	x/x	x/x	x/-

**Table 7:** Details of the malformations detected in C6 and C7 using the grading system presented above and statistical comparisons between body sides using LR chi square tests and Cramer’s V.

Cervical Vertebrae	Grade 0 *n* (%)	Grade 1 *n* (%)	Grade 2 *n* (%)	Grade 3 *n* (%)	Grade 4 *n* (%)	*p*-Value Cramer’s V
C6 (*n* = 39)						
Right	11 (28.2)	0	0	0 (0)	28 (71.8)	0.046
Left	3 (7.7)	0	1 (2.6)	1 (2.6)	34 (87.2)	0.460
Bilateral	2 (2.6)	0	0	0 (2.6)	27 (69.2)	
C7 (*n* = 39)						
Right	11 (28.2)	2 (5.1)	12 (30.8)	14 (35.9)	na	0.010
Left	3 (7.7)	1 (2.6)	13 (33.3)	22 (56.4)	na	0.411
Bilateral	2 (5.1)	0	8 (20.5)	13 (33.3)	na	

na: not applicable.

**Table 8:** Conditional distributions of the malformations detected in C6 and C7 classifying normal and malformed cervical vertebrae and statistical comparisons between normal and malformed cervical vertebrae (C6 versus C7) for each body side and both sides combined using LR chi square tests and Cramer’s V.

C6 (*n* = 39)	Normal	Normal	Malformed	Malformed	*p*-Value	Cramer’s V
C7 (*n* = 39)	Normal	Malformed	Normal	Malformed		
	*n* (%)	*n* (%)	*n* (%)	*n* (%)		
Right	10 (25.1)	1 (2.6)	1 (2.6)	27 (69.2)	<0.0001	0.873
Left	3 (7.7)	0	0	36 (92.3)	<0.0001	1.000
Both sides	2 (5.1)	0	0	37 (94.9)	<0.0001	1.000

**Table 9:** Details of the malformations detected in the first ribs using the grading system presented above and statistical comparisons between body sides using LR chi square tests and Cramer’s V. Missing ribs are scored with grade 4 for both conditions, length and width.

First Rib	Grade 1 *n* (%)	Grade 2 *n* (%)	Grade 3 *n* (%)	Grade 4 *n* (%)	Bifid *n* (%)	Normal *n* (%)	*p*-Value Cramer’s V
Length (*n* = 35)							
Right	1 (2.9)	3 (8.6)	15 (42.9)	2 (5.7)	2 (5.7)	12 (34.3)	0.194
Left	2 (5.7)	6 (17.1)	15 (42.9)	3 (8.6)	1 (2.9)	8 (22.9)	0.491
Bilateral	0 (2.3)	0 (15.8)	10 (28.6)	2 (5.7)	0	4 (11.4)	
Width (*n* = 37)							
Right	4 (10.8)	1 (2.7)	18 (48.7)	2 (5.4)	2 (5.4)	10 (27.0)	0.061
Left	3 (8.1)	2 (5.4)	23 (62.2)	3 (8.1)	1 (2.7)	9 (23.7)	0.559
Bilateral	0	1 (2.7)	13 (35.1)	2 (5.4)	0 (2.6)	0	

**Table 10:** Conditional distributions of the malformations detected in C6, C7 and the length of first ribs classifying normal and malformed cervical vertebrae and first ribs and statistical comparisons for each body side and both sides combined using LR chi square tests and Cramer’s V.

Vertebra	Normal	Normal	Malformed	Malformed	*p*-Value	Cramer’s V
First Rib Length	Normal	Malformed	Normal	Malformed		
	*n* (%)	*n* (%)	*n* (%)	*n* (%)		
C6						
Right (*n* = 38)	5 (13.2)	6 (15.8)	8 (21.1)	19 (50.0)	0.356	0.151
Left (*n* = 36)	1 (2.8)	2 (5.6)	7 (19.4)	26 (72.3)	0.644	0.081
Both sides (*n* = 38)	0	2 (5.6)	4 (10.5)	32 (84.2)	0.499	−0.081
C7						
Right (*n* = 38)	5 (13.2)	6 (15.8)	8 (21.1)	19 (50.0)	0.356	0.151
Left (*n* = 36)	1 (2.8)	2 (5.6)	7 (19.4)	26 (72.3)	0.644	0.081
Both sides (*n* = 38)	0	2 (5.6)	4 (10.5)	32 (84.2)	0.499	−0.081

**Table 11:** Conditional distributions of the malformations detected in C6, C7, the first and second ribs classifying normal and malformed cervical vertebrae, first and second ribs and statistical comparisons for each body side and both sides combined using LR chi square tests and Cramer’s V.

C6	Normal	Normal	Normal	Malformed	Malformed
C7	Normal	Normal	Malformed	Malformed	Malformed
First Rib Length	Normal	Malformed	Normal	Normal	Malformed
Second Rib	*n* (%)	*n* (%)	*n* (%)	*n* (%)	*n* (%)
Right (*n* = 33)					
Normal	4 (12.1)	1 (3.0)	1 (3.0)	5 (15.2)	1 (3.0)
Grade 1	0	4 (12.1)	0	0	13 (39.4)
Grade 2	0	0	0	0	2 (6.5)
Bifid	0	0	0	0	2 (6.5)
Total	4 (12.1)	5 (15.2)	1 (3.0)	5 (15.2)	18 (54.5)
Left (*n* = 31)					
Normal	0	1 (3.2)	0	4 (12.9)	2 (6.5)
Grade 1	0	1 (3.2)	0	2 (6.5)	16 (51.6)
Grade 2	0	0	0	0	4 (12.9)
Bifid	0	0	0	0	1 (3.2)
Total	0	2 (6.5)	0	6 (19.4)	23 (74.2)

**Table 12:** Estimates of relative variance proportions and correlations between the grades of C6, C7, first rib length, first rib width and second rib width from the generalized mixed model for correlated random coefficients.

Trait	C6	C7	First Rib Length	First Rib Width	Second Rib Width
C6	0.256 *	0.555 ***	0.357 *	0.278	0.140
C7		0.171	0.299 *	0.256 *	0.153
First rib length			0.336 *	0.883 ***	0.673 ***
First rib width				0.236	0.636 ***
Second rib width					0.194

*: *p* < 0.05, ***: *p* < 0.001.

## Data Availability

The data presented in this study are available upon request from the corresponding author.

## Supplementary Materials

The following supporting information can be downloaded at: https://www.mdpi.com/article/10.3390/ani13233732/s1, Table S1: Joint distributions of the different grades of C6 and C7 for the right and left side and for contralateral sides; Table S2: Joint distributions of normal and malformed C6 and C7 for the right and left side and for contralateral sides. All grades > 0 by side are encoded as 1 and normal vertebrae (grade 0) by side as 0; Table S3: Joint distributions of the different grades of the length and width of the first ribs for the right and left side and for contralateral sides; Table S4: Joint distributions of normal and malformed first ribs for the right and left side and for contralateral sides. All grades > 0 by side are encoded as 1 and normal ribs (grade 0) by side as 0; Table S5: Joint distributions of the different grades of the width of the second ribs for the right and left side; Table S6: Joint distributions of the different grades of the length of the first ribs for the right and left side with the width of the second ribs; Table S7: Joint distributions of the different grades of the width of the first ribs for the right and left side with the width of the second ribs.
