# Supplementary material for: Radiological Methods for the Imaging of Congenital Malformations of C6-T1, the First and Second Sternal Ribs and Development of a Classification System, Demonstrated in Warmblood Horses
Source: Animals (Basel). 2023 Dec 2;13(23):3732. doi: 10.3390/ani13233732 (PMC10705149; doi:10.3390/ani13233732)
Supplement: Supplementary file 1 [file animals-13-03732-s001.zip › animals-2642643-supplementary.pdf]

Table S1. Joint distributions of the different grades of C6 and C7 for the right and left side and for contralateral sides.

**Cervical vertebrae**		**C6-grades**					
**C6-right**		Grade 0	Grade 1	Grade 2	Grade 3	Grade 4	All
		11	0	0	0	28	39
**C7-right**	Grade 0	10	0	0	0	1	11
	Grade 1	1	0	0	0	1	2
	Grade 2	0	0	0	0	12	12
	Grade 3	0	0	0	0	14	14
	All	11	0	0	0	28	39
**C6-right**							
**C7-left**	Grade 0	2	0	0	0	1	3
	Grade 1	0	0	0	0	1	1
	Grade 2	4	0	0	0	9	13
	Grade 3	5	0	0	0	17	22
	All	11	0	0	0	28	39
**C6-left**		Grade 0	Grade 1	Grade 2	Grade 3	Grade 4	All
		3	0	1	1	34	39
**C7-left**	Grade 0	3	0	0	0	0	3
	Grade 1	0	0	0	0	1	1
	Grade 2	0	0	1	1	11	13
	Grade 3	0	0	0	0	22	22
	All	3	0	1	1	34	39
**C6-left**							
**C7-right**	Grade 0	2	0	1	1	7	11
	Grade 1	0	0	0	0	2	2
	Grade 2	1	0	0	0	11	12
	Grade 3	0	0	0	0	14	14
	All	3	0	1	1	34	39

Table S2. Joint distributions of normal and malformed C6 and C7 for the right and left side and for contralateral sides. All grades > 0 by side are encoded as 1 and normal vertebrae (grade 0) by side as 0.

**Cervical vertebrae**		**C6**		
**C6-right**		Normal	Malformed	All
		11	28	39
**C7-right**	Normal	10	1	11
	Malformed	1	27	28
	All	11	28	39
**C6-right**				
**C7-left**	Normal	2	1	3
	Malformed	9	27	36
	All	11	28	39
**C6-left**		Normal	Malformed	All
		3	36	39
**C7-left**	Normal	3	0	3
	Malformed	0	36	36
	All	3	36	39
**C6-left**				
**C7-right**	Normal	2	9	11
	Malformed	1	27	29
	All	3	36	39

Table S3. Joint distributions of the different grades of the length and width of the first ribs for the right and left side and for contralateral sides.

**Length of first rib**		**Length-grades**						
**R1-right**		Grade 0	Grade 1	Grade 2	Grade 3	Grade 4	Bifid	All
		12	1	3	15	2	2	35
**R1-left**	Grade 0	4	0	2	2	0	0	8
	Grade 1	2	0	0	0	0	0	2
	Grade 2	2	1	0	2	0	1	6
	Grade 3	3	0	1	10	0	1	15
	Grade 4	0	0	0	1	2	0	3
	Bifid	1	0	0	0	0	0	1
**Width of first rib**		**Width-grades**						
**R1-right**		Grade 0	Grade 1	Grade 2	Grade 3	Grade 4	Bifid	All
		10	4	1	18	2	2	37
**R1-left**	Grade 0	0	1	4	0	0	0	5
	Grade 1	3	0	0	0	0	0	3
	Grade 2	1	0	1	0	0	0	2
	Grade 3	5	3	0	13	0	2	23
	Grade 4	0	0	0	1	2	0	3
	Bifid	1	0	0	0	0	1	1
**Width of first rib**		**Width-grades**						
**R1-right**		Grade 0	Grade 1	Grade 2	Grade 3	Grade 4	Bifid	All
		10	4	0	20	2	2	38
**Length of R1-right**	Grade 0	10	3	0	0	0	0	13
	Grade 1	1	0	0	0	0	0	1
	Grade 2	0	0	0	3	0	0	3
	Grade 3	0	0	0	17	0	0	17
	Grade 4	0	0	0	0	2	0	2
	Bifid	0	0	0	0	0	2	2
**Width of first rib**	**Width-grades**							
**R1-right**		Grade 0	Grade 1	Grade 2	Grade 3	Grade 4	Bifid	All
		9	4	1	18	2	2	36
**Length of R1-left**	Grade 0	3	1	0	4	0	0	8
	Grade 1	2	0	0	0	0	0	2
	Grade 2	1	2	0	2	0	1	6
	Grade 3	2	1	2	11	0	1	16
	Grade 4	0	0	0	1	2	0	3
	Bifid	1	0	0	0	0	0	1

**Width of first rib**	**Width-grades**							
**R1-left**		Grade 0	Grade 1	Grade 2	Grade 3	Grade 4	Bifid	All
		5	3	1	23	3	1	36
**Length of R1-right**	Grade 0	1	3	1	7	0	1	13
	Grade 1	0	0	0	1	0	0	1
	Grade 2	2	0	0	1	0	0	3
	Grade 3	2	0	0	12	1	0	15
	Grade 4	0	0	0	0	2	0	2
	Bifid	0	0	0	2	0	0	2
**Width of first rib**	**Width-grades**							
**R1-left**		Grade 0	Grade 1	Grade 2	Grade 3	Grade 4	Bifid	All
		5	3	2	22	3	1	36
**Length of R1-left**	Grade 0	5	2	1	0	0	0	8
	Grade 1	0	1	0	1	0	0	2
	Grade 2	0	0	0	6	0	0	6
	Grade 3	0	0	1	15	0	0	16
	Grade 4	0	0	0	0	3	0	3
	Bifid	0	0	0	0	0	1	1

Table S4. Joint distributions of normal and malformed first ribs for the right and left side and for contralateral sides. All grades > 0 by side are encoded as 1 and normal ribs (grade 0) by side as 0.

**First ribs**		**Length**		
**Length of R1-right**		Normal	Malformed	All
		12	23	35
**Length of R1-left**	Normal	4	4	8
	Malformed	8	19	27
		**Width**		
**Width of R1-right**		Normal	Malformed	All
		10	27	37
**Width of R1-left**	Normal	0	5	5
	Malformed	10	22	32
		**Width**		
**Width of R1-right**		Normal	Malformed	All
		10	28	38
**Length of R1-right**	Normal	10	3	13
	Malformed	0	25	25
		**Width**		
**Width of R1-right**		Normal	Malformed	All
		9	27	36
**Length of R1-left**	Normal	3	5	8
	Malformed	6	22	28
		**Width**		
**Width of R1-left**		Normal	Malformed	All
		5	31	36
**Length of R1-right**	Normal	1	12	13
	Malformed	4	19	23
		**Width**		
**Width of R1-left**		Normal	Malformed	All
		5	31	36
**Length of R1-left**	Normal	5	3	8
	Malformed	0	28	28

Table S5. Joint distributions of the different grades of the width of the second ribs for the right and left side.

**Width of second rib**	**Width-grades**							
**R2-right**		Grade 0	Grade 1	Grade 2	Grade 3	Grade 4	Bifid	All
		12	15	2	0	0	2	31
**Width of R2-left**	Grade 0	4	3	0	0	0	0	7
	Grade 1	7	10	0	0	0	2	19
	Grade 2	0	2	2	0	0	0	4
	Grade 3	0	0	0	0	0	0	0
	Grade 4	0	0	0	0	0	0	0
	Bifid	1	0	0	0	0	0	1

Table S6. Joint distributions of the different grades of the length of the first ribs for the right and left side with the width of the second ribs.

**Length of first rib**		**Length-grades**						
**R1-right**		Grade 0	Grade 1	Grade 2	Grade 3	Grade 4	Bifid	All
		10	1	3	16	1	2	33
**Width of R2-right**	Grade 0	10	0	0	2	0	0	12
	Grade 1	0	1	2	13	1	0	17
	Grade 2	0	0	1	1	0	0	2
	Grade 3	0	0	0	0	0	0	0
	Grade 4	0	0	0	0	0	0	0
	Bifid	0	0	0	0	0	2	2
**Length of first rib**		**Length-grades**						
**R1-right**		Grade 0	Grade 1	Grade 2	Grade 3	Grade 4	Bifid	All
		10	1	3	14	0	2	30
**Width of R2-left**	Grade 0	2	0	2	3	0	0	7
	Grade 1	7	0	0	9	0	2	18
	Grade 2	0	1	1	2	0	0	4
	Grade 3	0	0	0	0	0	0	0
	Grade 4	0	0	0	0	0	0	0
	Bifid	1	0	0	0	0	0	1
**Length of first rib**		**Length-grades**						
**R1-left**		Grade 0	Grade 1	Grade 2	Grade 3	Grade 4	Bifid	All
		6	2	6	15	2	1	32
**Width of R2-right**	Grade 0	3	2	2	4	0	1	12
	Grade 1	3	0	3	8	2	0	16
	Grade 2	0	0	0	2	0	0	2
	Grade 3	0	0	0	0	0	0	0
	Grade 4	0	0	0	0	0	0	0
	Bifid	0	0	1	1	0	0	2
**Length of first rib**		**Length-grades**						
**R1-left**		Grade 0	Grade 1	Grade 2	Grade 3	Grade 4	Bifid	All
		6	2	6	15	1	1	31
**Width of R2-left**	Grade 0	4	1	0	2	0	0	7
	Grade 1	2	1	5	10	1	0	19
	Grade 2	0	0	1	3	0	0	4
	Grade 3	0	0	0	0	0	0	0
	Grade 4	0	0	0	0	0	0	0
	Bifid	0	0	0	0	0	1	1

Table S7. Joint distributions of the different grades of the width of the first ribs for the right and left side with the width of the second ribs.

**Width of first rib**		**Width-grades**						
**R1-right**		Grade 0	Grade 1	Grade 2	Grade 3	Grade 4	Bifid	All
		8	3	1	19	1	2	34
**Width of R2-right**	Grade 0	8	2	0	2	0	0	12
	Grade 1	0	1	1	15	1	0	18
	Grade 2	0	0	0	2	0	0	2
	Grade 3	0	0	0	0	0	0	0
	Grade 4	0	0	0	0	0	0	0
	Bifid	0	0	0	0	0	2	2
**Width of first rib**		**Width-grades**						
**R1-right**		Grade 0	Grade 1	Grade 2	Grade 3	Grade 4	Bifid	All
		8	3	1	17	0	2	31
**Width of R2-left**	Grade 0	2	0	0	5	0	0	7
	Grade 1	5	2	1	9	0	2	19
	Grade 2	0	1	0	3	0	0	4
	Grade 3	0	0	0	0	0	0	0
	Grade 4	0	0	0	0	0	0	0
	Bifid	1	0	0	0	0	0	1
**Width of first rib**	**Width-grades**							
**R1-left**		Grade 0	Grade 1	Grade 2	Grade 3	Grade 4	Bifid	All
		3	3	2	21	2	1	32
**Width of R2-right**	Grade 0	0	3	1	7	0	1	12
	Grade 1	3	0	1	10	2	0	16
	Grade 2	0	0	0	2	0	0	2
	Grade 3	0	0	0	0	0	0	0
	Grade 4	0	0	0	0	0	0	0
	Bifid	0	0	0	2	0	0	2
**Width of first rib**	**Width-grades**							
**R1-left**		Grade 0	Grade 1	Grade 2	Grade 3	Grade 4	Bifid	All
		3	3	2	21	1	1	31
**Width of R2-left**	Grade 0	3	1	0	3	0	0	7
	Grade 1	0	2	2	14	1	0	19
	Grade 2	0	0	0	4	0	0	4

	Grade 3	0	0	0	0	0	0	0
	Grade 4	0	0	0	0	0	0	0
	Bifid	0	0	0	0	0	1	1
